# Reducing central serotonin in adulthood promotes hippocampal neurogenesis

**DOI:** 10.1038/srep20338

**Published:** 2016-02-03

**Authors:** Ning-Ning Song, Yun-Fang Jia, Lei Zhang, Qiong Zhang, Ying Huang, Xiao-Zhen Liu, Ling Hu, Wei Lan, Ling Chen, Klaus-Peter Lesch, Xiaoyan Chen, Lin Xu, Yu-Qiang Ding

**Affiliations:** 1 Key Laboratory of Arrhythmias, Ministry of Education, East Hospital, Tongji University School of Medicine, Shanghai, China; 2 Department of Anatomy and Neurobiology, Collaborative Innovation Center for Brain Science, Tongji University School of Medicine, Shanghai, China; 3 Key Laboratory of Animal Models and Human Disease Mechanisms of the Chinese Academy of Sciences & Yunnan Province, Kunming Institute of Zoology, Kunming, Yunnan, China; 4 Graduate University of the Chinese Academy of Sciences, Beijing, China; 5 Department of Human Anatomy, Guangxi Medical University, Nanning, Guangxi, China; 6 Division of Molecular Psychiatry, Department of Psychiatry and Psychotherapy, University of Würzburg, Würzburg, Germany; 7 Shanghai Institute of Materia Medica, Chinese Academy of Sciences, Shanghai, China

## Abstract

Chronic administration of selective serotonin reuptake inhibitors (SSRIs), which up-regulates central serotonin (5-HT) system function, enhances adult hippocampal neurogenesis. However, the relationship between central 5-HT system and adult neurogenesis has not fully been understood. Here, we report that lowering 5-HT level in adulthood is also able to enhance adult hippocampal neurogenesis. We used tamoxifen (TM)-induced Cre in *Pet1*-CreER^T2^ mice to either deplete central serotonergic (5-HTergic) neurons or inactivate 5-HT synthesis in adulthood and explore the role of central 5-HT in adult hippocampal neurogenesis. A dramatic increase in hippocampal neurogenesis is present in these two central 5-HT-deficient mice and it is largely prevented by administration of agonist for 5-HTR2c receptor. In addition, the survival of new-born neurons in the hippocampus is enhanced. Furthermore, the adult 5-HT-deficient mice showed reduced depression-like behaviors but enhanced contextual fear memory. These findings demonstrate that lowering central 5-HT function in adulthood can also enhance adult hippocampal neurogenesis, thus revealing a new aspect of central 5-HT in regulating adult neurogenesis.

Two major regions of the rodent brain undergo continuous neurogenesis into adulthood: the subventricular zone, from where newly-generated neurons migrate to the olfactory bulb, and the subgranular zone (SGZ), which gives rise to new dentate gyrus granular neurons^1^,^2^,^3^. Adult hippocampal neurogenesis is controlled by many factors, including a number of neurotransmitters^3^,^4^. The neurotransmitter serotonin (5-HT) is synthesized in the brain by the enzyme tryptophan hydroxylase 2 (Tph2)^5^. After its release into the synaptic cleft, 5-HT is removed by the presynaptic 5-HT transporter (5-HTT)^6^. Monoamine system dysfunction is believed to be a major causative factor of depression^7^. For this reason, SSRIs are frequently used as first-line antidepressants, in an effort to enhance central 5-HT system function.

Chronic administration of the SSRI fluoxetine leads to increased hippocampal neurogenesis in adult mice^8^. The role of 5-HT in adult hippocampal neurogenesis has also been investigated in various genetic mouse models, but results were somewhat different than expected. *5-HTT*^−/−^ mice showed elevated 5-HT level in the brain, but an increase of proliferation of adult hippocampal stem cells is only observed in aged*5-HTT*^−/−^ mice (>14.5 months) but not younger adult mice (3 weeks and 3 months)^9^. *Tph2*^−/−^ mice that lack 5-HT in the brain, however, present a phenotype similar to that of *5-HTT*^−/−^ mice^10^. In addition to the proliferation of the stem cells, chronic SSRI administration also enhances the survival of adult-born neurons in the hippocampus^11^. On the other hand, the increased survival of adult-born neurons has been reported recently in the genetic mouse model with less central 5-HT^12^,^13^. Thus, the relationship between central 5-HT and adult hippocampal neurogenesis including proliferation of neural stem cells and survival of new-born neurons has not fully been understood.

5-HT is implicated in several aspects of nervous system development, including axonal growth and dendritic spine formation, as well as barrel formation and synaptic plasticity in the somatosensory cortex^14^,^15^,^16^. It is therefore likely that deleting either Tph2, 5-HT receptors (5-HTR) or 5-HTT by means of conventional gene targeting might impair brain development and lead to uncontrolled pleiotropic effects, particularly since most of these genes are already expressed during embryonic stages. To circumvent these potential complications and allow the brain to first develop normally, we used *Pet1*-CreER^T2^; Rosa26-DTA (diphtheria fragment A) mice to deplete central 5-HTergic neurons, and *Pet1*-CreER^T2^; *Tph2*^flox/flox^ mice to inactivate central 5-HT synthesis, both in adulthood. We found that adult neurogenesis is significantly increased in the SGZ, thus revealing an unexpected role for central 5-HT in regulating adult neurogenesis.

## Results

### Depletion of central 5-HTergic neurons in adulthood

**Figure 1:**
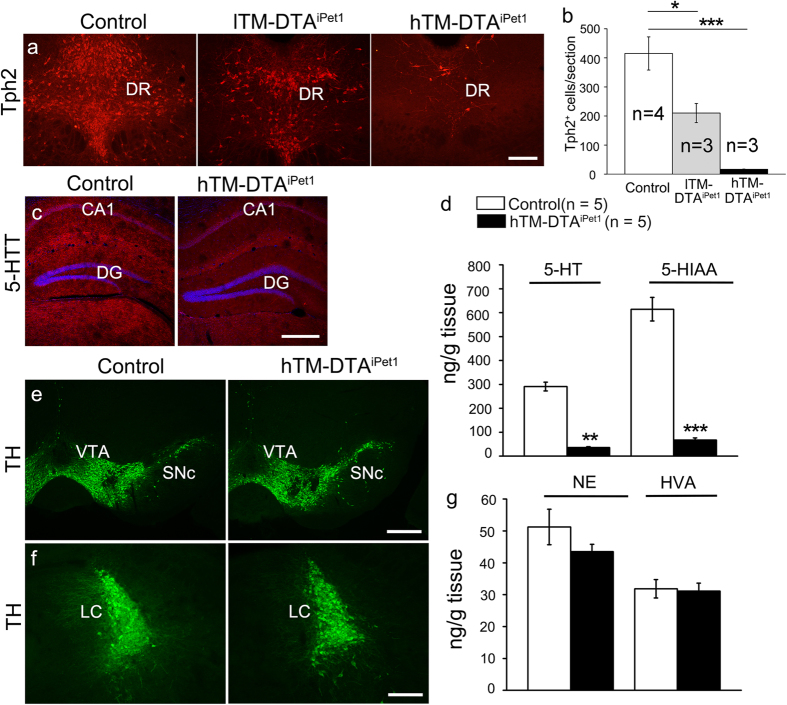
Depletion of central 5-HTergic neurons in DTA^iPet1^ mice. **(a,b)** 5-HTergic neurons are depleted in the dorsal raphe nucleus (DR) after administration of TM in adult DTA^iPet1^ mice. Tph2 immunostaining reveals that approximately half of 5-HTergic neurons remain in lTM-DTA^iPet1^ mice (80 mg/kg, once daily for 3 days), but only 5% of 5-HTergic neurons are present in hTM-DTA^iPet1^ mice (250 mg/kg, once daily for 4 days). The number of Tph2^+^ neurons in the DR of each of the three treatment groups is quantified in **(b)**, using one-way ANOVA (alpha = 0.05) with *post hoc* Tukey test (*p* = 0.025 for comparison of control and lTM-DTA^iPet1^, *p* = 0.001 for comparison of control and hTM-DTA^iPet1^). **(c)** 5-HTT^+^ fibers (red) are abundantly distributed throughout the hippocampus of control mice, but only a very few of them are observed in hTM-DTA^iPet1^ mice. **(d)** HPLC assay shows hTM-DTA^iPet1^ mice have extremely low levels of 5-HT and its metabolite 5-HIAA comparing to control mice. Data of 5-HT level were compared using Mann-Whitney rank sum test (*p* = 0.008), and data of 5-HIAA using Student’s t-test (*p* = 4.5 × 10^−6^). **(e,f)** Immunostaining of TH shows similar expression in midbrain dopaminergic neurons **(e)** and hindbrain norepinephrinergic neurons **(f)** between control and hTM-DTA^iPet1^ mice. **(g)** HPLC analysis shows comparable levels of HVA, a metabolite of dopamine, and NE in control and hTM-DTA^iPet1^ mice. Data were compared using Student’s *t*-test.CA1, field CA1 of hippocampus; DG, dentate gyrus; DR, dorsal raphe; 5-HIAA, 5-Hydroxyindoleacetic acid; HVA, homovanillic acid; LC, locus coeruleus; NE, norepinephrine; SNc, substantia nigra, compact part; VTA, ventral tegmental area. All data are presented as mean ± s.e.m. *p < 0.05; **p < 0.01; ***p < 0.001. Scale bars represent 200 μm **(a)**, 400 μm **(c)**, 500 μm **(e)** and 200 μm **(f)**.

To specifically ablate 5-HTergic neurons in the adult brain, we crossed *Pet1*-CreER^T2^ mice, in which inducible Cre recombinase is selectively activated in 5-HTergic neurons^17^, with Rosa26-DTA mice^18^ to generate *Pet1*-CreER^T2^; Rosa26-DTA (referred to as DTA^iPet1^) offspring. TM administration led to the selective depletion of 5-HTergic neurons in the brainstem of DTA^iPet1^ mice, due to the toxic effect of DTA. One month after the final TM treatment, mice were sacrificed for analysis. When a lower dose of TM (80 mg/kg body weight, once daily for 3 consecutive days) was used, approximately 50% of 5-HTergic neurons were depleted, as illustrated by the striking decrease in the number of Tph2^+^ neurons in the dorsal raphe nucleus (Fig. 1a,b); these mice were referred to as lTM-DTA^iPet1^ mice. On the other hand, when a higher dose of TM (250 mg/kg body weight, once daily for 4 consecutive days) was used, more than 95% of 5-HTergic neurons were depleted (Fig. 1a,b); these mice were referred to as hTM-DTA^iPet1^ mice. Correspondingly, only a few 5-HTergic fibers were observed in the hippocampus, as shown by 5-HTT immunostaining (Fig. 1c). HPLC showed that 5-HT and its metabolite 5-HIAA were dramatically decreased in hTM-DTA^iPet1^ mice (Fig. 1d), which indicated that central 5-HT was dramatically reduced as a result of brainstem 5-HTergic neuronal loss. To determine whether other monoamines were affected or not after loss of 5-HTergic neurons, we examined the expression of tyrosine hydroxylase (TH), which labels dopaminergic neurons in the midbrain (Fig. 1e) and norepinephrinergic neurons in the hindbrain (Fig. 1f), respectively. We found that TH expression in hTM-DTA^iPet1^ mice was comparable to that in control mice. In consistent with these immunostaining data, HPLC showed that both the concentration of norepinephrine and homovanillic acid, the metabolite of dopamine, was not altered in hTM-DTA^iPet1^ mice relative to that of control mice (Fig. 1g). Together, 5-HTergic neurons in the brain can be selectively depleted in TM-DTA^iPet1^ mice in adulthood.

### A drastic increase of immature neurons in hTM-DTA^iPet1^ mice

**Figure 2:**
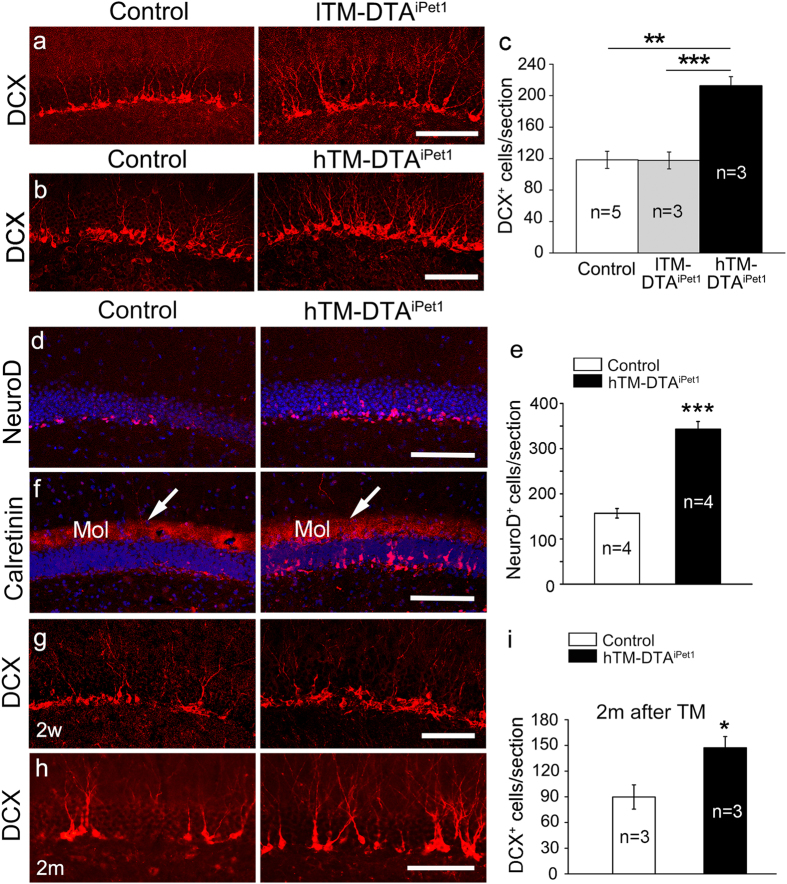
Enhanced adult hippocampal neurogenesis in hTM-DTA^iPet1^ mice. **(a–c)** The number of DCX^+^ cells in the SGZ is dramatically increased in hTM-DTA^iPet1^ mice **(b)**, but not in lTM-DTA^iPet1^ mice **(a)**. A quantification of DCX^+^ cells in the SGZ is shown in **(c)**, using one-way ANOVA (alpha = 0.05) with *post hoc* Tukey test (*p* = 0.002 for comparison of control and hTM-DTA^iPet1^, *p* = 9.8 × 10^−4^ for comparison of lTM-DTA^iPet1^and hTM-DTA^iPet1^). **(d,e)** The number of NeuroD^+^ cells is significantly increased in hTM-DTA^iPet1^ mice. Data were compared using Student’s *t*-test (*p* = 8.8 × 10^-5^). **(f)** Immunohistochemical analysis reveals that CR^+^ neurons are greatly increased in the hippocampal SGZ of hTM-DTA^iPet1^ mice relative to controls. No changes of CR^+^ hilar mossy neuron projections in the molecular layer of DG (arrows). **(g)** Two weeks after the last TM injection, the number of DCX^+^ cells is increased in the SGZ of hTM-DTA^iPet1^ mice. **(h,i)** Two months after the last TM injection, the number of DCX^+^ cells remains significantly increased in the SGZ of hTM-DTA^iPet1^ mice. Data were compared using Student’s *t*-test (*p* = 0.04). All data are presented as mean ± s.e.m. *p < 0.05; **p < 0.01; ***p < 0.001. Scale bars represent 150 μm (**a,d,f**) and 100 μm (**b,g,h**).

Adult neurogenesis involves proliferation of neural stem cells and differentiation of new-born neurons, two processes that require coordinated expression of a number of different genes^3^. Firstly, to detect the change of immature neurons after loss of 5-HTergic neurons, DCX immunostaining was performed to label the neuroblasts and immature neurons in the SGZ. We found that the number of DCX-labeled cells in lTM-DTA^iPet1^ mice was comparable to that in control mice (Fig. 2a,c), indicating that overall neurogenesis level in the hippocampus was unaffected in lTM-DTA^iPet1^ mice. In contrast to lTM-DTA^iPet1^ mice, the number of DCX^+^ cells in the SGZ was dramatically increased in hTM-DTA^iPet1^ mice (Fig. 2b,c). In addition to DCX^+^ cells, the number of NeuroD^+^ neuroblasts was also significantly increased in hTM-DTA^iPet1^ mice (Fig. 2d,e). Furthermore, the number of CR^+^, which is expressed in the immature neurons following the initiation of DCX expression^1^, were increased also in hTM-DTA^iPet1^ mice (Fig. 2f). There was no change of CR^+^ fibers in the molecular layer (arrows, Fig. 2f), which are originated from hilar mossy neurons. Finally, the enhanced hippocampal neurogenesis was first detected two weeks after the last TM injection (Fig. 2g) and also observed two months after the last TM injection (Fig. 2h,i), demonstrating that this effect was not a transient one. Together, these results indicate that removing central 5-HTergic neurons during adulthood leads to a significant enhancement in the hippocampal neurogenesis.

### Proliferation of neural stem cells is enhanced in hTM-DTA^iPet1^ mice

**Figure 3:**
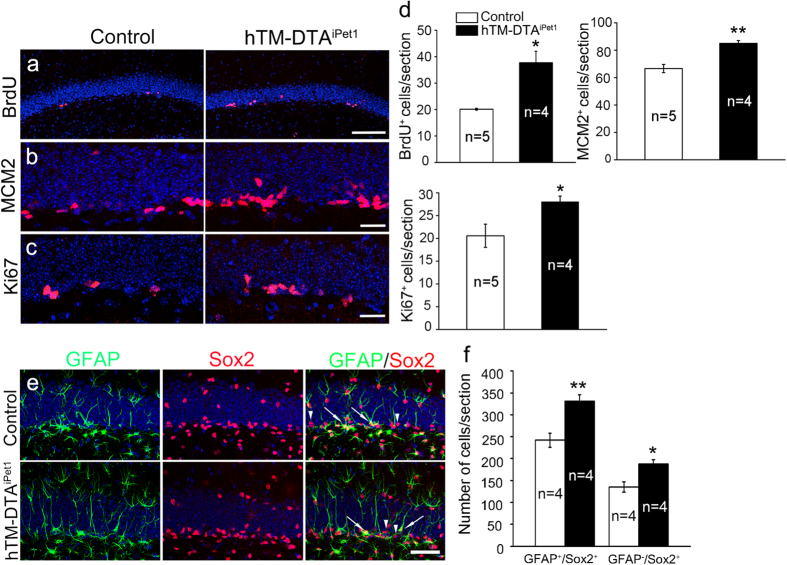
Proliferation of neural stem cells is enhanced in hTM-DTA^iPet1^ mice. **(a)** The number of BrdU^+^ cells (red) in the SGZ is dramatically increased in hTM-DTA^iPet1^ mice. Hoechst counterstaining is shown in blue. **(b,c)** The number of MCM2^+^ (red in **b**) and Ki67^+^ cells (red in **c)** in the SGZ is significantly increased in hTM-DTA^iPet1^ mice. Hoechst counterstaining is shown in blue. **(d)** Quantification of data in **(a–c).** Data of BrdU^+^ cell were compared using Mann-Whitney rank sum test (*p* = 0.016), and others using Student’s *t*-test (*p* = 0.002 for MCM2^+^ cells, *p* = 0.045 for Ki67^+^ cells).**(e, f)** The numbers of GFAP^+^/Sox2^+^ cells (QNPs, arrows) and GFAP^−^/Sox2^+^ cells (ANPs, arrowheads) are both significantly increased in the SGZ of hTM-DTA^iPet1^ mice relative to controls. Data were compared using Student’s *t*-test (*p* = 0.004 for GFAP^+^/Sox2^+^ cells, *p* = 0.013 for GFAP^-^/Sox2^+^ cells). All data are presented as mean ± s.e.m. *p < 0.05; **p < 0.01. Scale bars represent 100 μm **(a)**, 25 μm **(b,c)** and 50 μm **(e)**.

To explore the cause of increase of neuroblasts and immature neurons after depleting 5-HTergic neurons, we checked the proliferation of adult stem cells in the SGZ. First, we examined neural stem cell proliferation by means of BrdU incorporation method, and found that the number of BrdU^+^ cells was dramatically increased in hTM-DTA^iPet1^ mice compared with controls (Fig. 3a,d). Besides, we also examined the expression of two other proteins, MCM2 and Ki67, which are expressed in cell cycles of neural stem cells^19^. Similar to BrdU^+^ cells, both MCM2^+^ and Ki67^+^ cells in hTM-DTA^iPet1^ mice were significantly increased (Fig. 3b–d). These results indicate that 5-HTergic neuron depletion leads to increased neural stem cell proliferation in the SGZ.

SSRIs enhance adult hippocampal neurogenesis by increasing the number of amplifying neural progenitor cells (ANPs) rather than quiescent neural progenitor cells (QNPs)^20^. To determine whether QNPs or ANPs were affected in hTM-DTA^iPet1^ mice, we performed GFAP and Sox2 co-immunostaining^1^ and found that the number of GFAP^+^/Sox2^+^ QNPs was significantly elevated in the SGZ of hTM-DTA^iPet1^ mice (Fig. 3e,f). A similar increase was also observed in GFAP^-^/Sox2^+^ ANPs (Fig. 3e,f). We therefore conclude that the enhanced adult hippocampal neurogenesis in hTM-DTA^iPet1^ mice is due to an increase in both QNPs and ANPs in the SGZ.

### Increased survival of new-born neurons in hTM-DTA^iPet1^ mice

**Figure 4:**
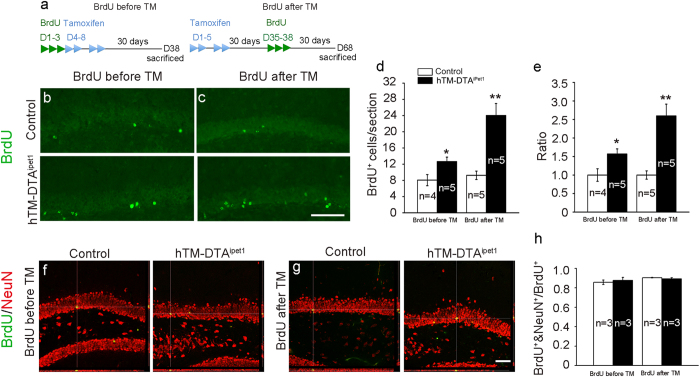
Survival of adult-born neurons is enhanced in hTM-DTA^iPet1^ mice. **(a)** Scheme of survival analysis. For studying the survival of neuron born before depleting 5-HTergic neurons (BrdU before TM), BrdU was injected for 3 consecutive days, followed by 4 times of TM administration. Mice were sacrificed 30 days after last TM administration. For studying the survival of neuron born after depleting 5-HTergic neurons (BrdU after TM), TM was administrated as above and BrdU was injected 30 days after TM administration. Mice were sacrificed 30 days after TM administration. **(b,d,e)** BrdU was injected before TM administration. More BrdU^+^ cells are labeled in hTM-DTA^iPet1^ mice relative to control. **(c–e)** BrdU was injected 30 days after TM administration. More BrdU^+^ cells are labeled in hTM-DTA^iPet1^ mice relative to control. Data of BrdU^+^ cells in mice with BrdU injected before TM administrationwere compared using Student’s *t*-test (*p* = 0.033 in **d** and **e**) and data of BrdU^+^ cells in mice with BrdU injected after TM administrationwere compared using Mann-Whitney rank sum test (*p* = 0.008 in **d** and **e**). **(f–h)** Double immunostaining of BrdU and NeuN was performed in both “BrdU before TM” and “BrdU after TM” groups. The ratios of BrdU^+^/NeuN^+^ to BrdU^+^ cells are comparable in both control and hTM-DTA^iPet1^ mice, no matter whether BrdU was incorporated before or after TM administration. Data of percentage were compared using Student’s *t*-test. All data are presented as mean ± s.e.m. *p < 0.05; **p < 0.01. Scale bars represent 100 μm in **(b,c)** and 50 μm in **(f,g)**.

Chronic SSRI administration enhances the survival of adult-born neurons^11^, and we next set out to explore whether the survival of adult-born neurons was affected or not after adult depletion of 5-HTergic neurons. Firstly, to detect the survival of new-born cells generated before depleting 5-HTergic neurons, BrdU was injected prior to TM administration (Fig. 4a). Because depleting 5-HTergic neurons only occurs after TM administration, BrdU could label similar numbers of proliferating cells in both hTM-DTA^iPet1^ and control mice, and BrdU^+^ cells in the hippocampus 30 days after TM administration could be used to reflect changes in the survival of adult-born neurons in hTM-DTA^iPet1^ mice. We found that BrdU^+^ cells in the SGZ of hTM-DTA^iPet1^ mice were increased to about 1.6 fold of that in control mice (Fig. 4b,d,e). Secondly, to determine the survival of new-born neurons generated after depleting 5-HTergic neurons, BrdU were injected 30 days after the TM treatment. BrdU^+^ cells in the SGZ of hTM-DTA^iPet1^ mice were increased to about 2.6 fold of that in control mice (Fig. 4c–e). To confirm whether BrdU-incorporated cells eventually become neurons or not, we performed BrdU/NeuN double immunostaining. We found that most of BrdU^+^cells were NeuN^+^ in both control and hTM-DTA^iPet1^ mouse brains without obvious differences (Fig. 4f–g). It is concluded that the survival of new-born neurons in the SGZ is enhanced in hTM-DTA^iPet1^ mice.

### Enhanced hippocampal neurogenesis is also present in adult Tph2^iPet1^ mice

**Figure 5:**
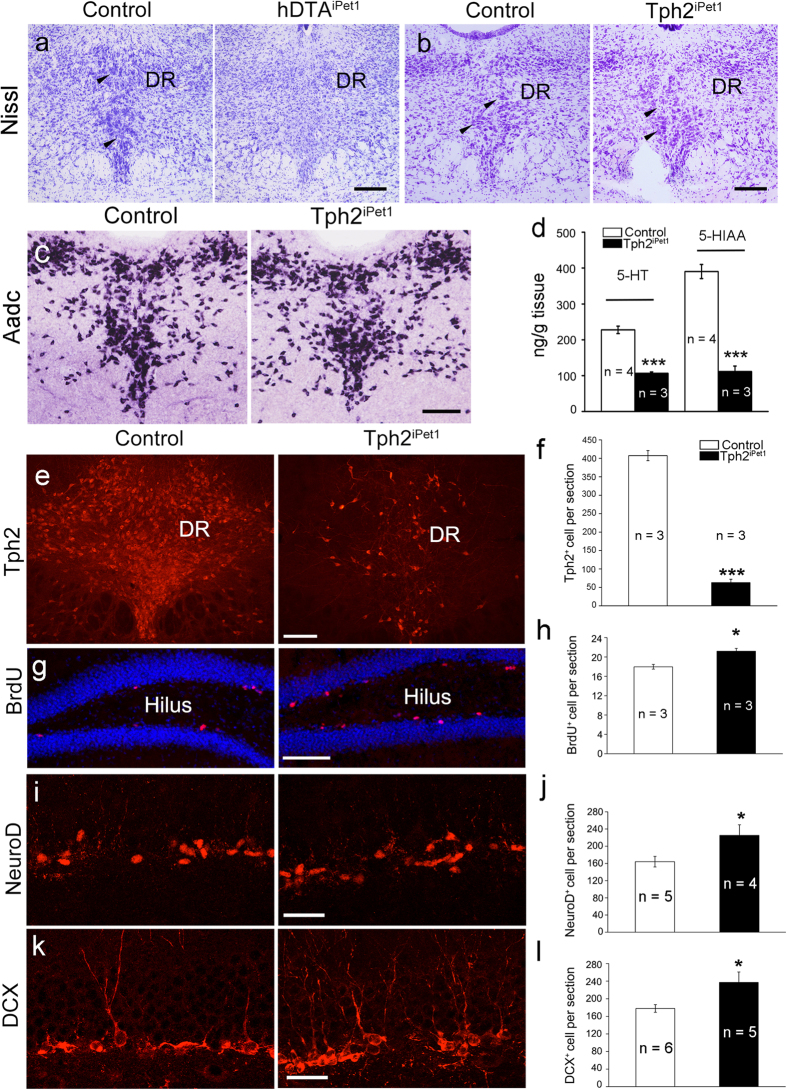
Enhanced adult hippocampal neurogenesis in Tph2^iPet1^ mice. **(a)** Nissl staining showing that intensely stained 5-HTergic neurons (arrowheads) are present in the DR of control mice, but absent in hTM-DTA^iPet1^ mice. **(b)** Nissl staining showing intensely stained 5-HTergic neurons (arrowheads) in the DR of Tph2^iPet1^ and control mice. **(c)** *In situ* hybridization showing comparable numbers of AADC^+^ cells in the dorsal raphe nucleus (DR) of Tph2^iPet1^ and control mice **(c)**. **(d)** HPLC data show that 5-HT and its metabolite 5-HIAA in Tph2^iPet1^ mice reduce dramatically comparing to control mice. Data were compared using Student’s *t*-test (*p* = 2.0 × 10^−4^ for 5-HT and *p* = 1.3 × 10^−4^ for 5-HIAA). **(e,f)** The number of Tph2^+^ neurons is dramatically decreased in Tph2^iPet1^ mice, to approximately 15% of control levels. Data were compared using Student’s *t*-test (*p* = 3.0 × 10^−5^). **(g,h)** The number of BrdU^+^ cell is significantly increased in Tph2^iPet1^ mice relative to controls. Data were compared using Student’s *t*-test (*p* = 0.013). **(i,j)** The number of NeuroD^+^ neuroblasts is also significantly increased in Tph2^iPet1^ mice relative to controls. Data were compared using Student’s *t*-test (*p* = 0.048). **(k,l)** The number of DCX^+^ cells is significantly increased in Tph2^iPet1^ mice compared with controls. Data were compared using Student’s *t*-test (*p* = 0.032). All data are presented as mean ± s.e.m. *p < 0.05; ***p < 0.001. Scale bars represent 200 μm (**a–c,e,g**) and 30 μm (**i,k**).

In hTM-DTA^iPet1^ mice, 5-HTergic neurons are depleted due to toxicity of DTA. To ascertain whether enhanced adult hippocampal neurogenesis is caused by the loss of central 5-HTergic neurons or loss of 5-HT itself, *Pet1*-CreER^T2^ mice were crossed with *Tph2*^flox/flox^ mice^21^, to create *Pet1*-CreER^T2^; *Tph2*^flox/flox^ (referred to as Tph2^iPet1^) mice in which 5-HTergic neurons are intact but 5-HT synthesis is inactivated upon TM injection. Nissl staining showed that intensely-stained “5-HTergic-like” neurons were present in the dorsal raphe nucleus of Tph2^iPet1^ mice (arrowheads, Fig. 5b), but absent from hTM-DTA^iPet1^ mice (Fig. 5a). The number of Tph2^+^ neurons remaining in Tph2^iPet1^ mice was reduced to approximately 15% of controls (Fig. 5e,f), and HPLC data also revealed a drastic reduction of 5-HT and 5-HIAA in Tph2^iPet1^ mice (Fig. 5d). The maintenance of 5-HTergic neurons in Tph2^iPet1^ mice was confirmed by *in situ* hybridization of aromatic L-amino acid decarboxylase (AADC; Fig. 5c), a marker for 5-HTergic neurons. Thus, 5-HTergic neurons remained in Tph2^iPet1^ brain, despite Tph2 inactivation in most central 5-HTergic neurons. Importantly, BrdU^+^, NeuroD^+^ and DCX^+^ cell numbers were also increased in the SGZ of Tph2^iPet1^ mice relative to controls (Fig. 5g–l), although to a lesser extent than that observed in hTM-DTA^iPet1^ mice. Taken together, these findings indicate that reducing central 5-HT levels in adulthood by inactivation of Tph2 is also capable of increasing adult hippocampal neurogenesis.

### Administration of 5-HTR2c agonist prevents the increased neurogenesis in hTM-DTA^iPet1^ mice

**Figure 6:**
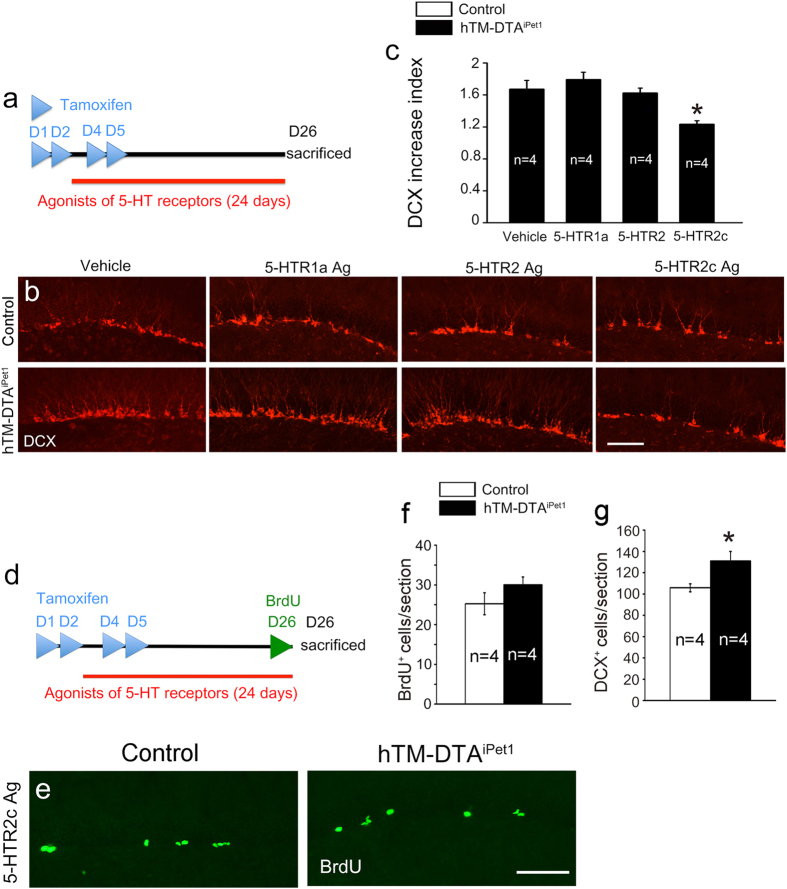
HTR2c agonist prevents the increase of adult hippocampal neurogenesis in hTM-DTA^iPet1^ mice. **(a)** Scheme of combinatorial injection of tamoxifen and 5-HTR agonists in mice. Tamoxifen was injected on day 1, 2, 4 and 5, while agonists was from day 3 to day 26. **(b)** Representative figures of DCX^+^ cells in control mice and hTM-DTA^iPet1^ mice treated with different agonists. **(c)** Quantification of DCX^+^ cells in control and hTM-DTA^iPet1^ mice treated with different agonists. Numbers of DCX^+^ cells in hTM-DTA^iPet1^ mice were normalized to those of control mice, and expressed as DCX increase index. 5-HTR1a and 5-HTR2 family agonists have no apparent effects on the increase of DCX^+^ cells, but 5-HTR2c agonist partially blocks this increase in hTM-DTA^iPet1^ mice. Data were compared using one-way ANOVA (alpha = 0.05) with *post hoc* Tukey test (*p* = 0.011 for comparison of vehicle and 5-HTR2c agonist). **(d)** Scheme of combinatorial injection of tamoxifen, 5-HTR agonists and BrdU in mice. **(e)** After injection of 5-HTR2c agonist, the increase of BrdU^+^ cells in hTM-DTA^iPet1^ mice is prevented. **(f,g)** Quantification of BrdU^+^ cells and DCX^+^ cells. There is no significant differences between control and hTM-DTA^iPet1^ mice in number of BrdU^+^ cells while more DCX^+^ cells are present in hTM-DTA^iPet1^ mice. Data were compared using Student’s *t*-test (*p* = 0.041 in **g**). All data are presented as mean ± s.e.m. *p < 0.05. Scale bars represent 100 μm **(b,e)**.

We speculate that the depletion of 5-HTergic neurons or lowering 5-HT level leads to impairments of activation of some 5-HTRs, which in turn results in the enhanced hippocampal neurogenesis. We therefore set out to explore whether administration of agonists of 5-HTRs in the hTM-DTA^iPet1^ mice could prevent the increased adult neurogenesis or not. As shown in Fig. 6a, both control and hTM-DTA^iPet1^ mice were injected with 8-OH DPAT, α-methyl-5-hydroxytryptamine maleate, or WAY161503, which are the agonists for 5-HTR1a, 5-HTR2 family and 5-HTR2c, respectively, for consecutive 24 days and were sacrificed for immunostaining of DCX. To screen out which receptor was involved, as the first step we used DCX increase index (normalizing the numbers of DCX^+^ cells in agonist-treated hTM-DTA^iPet1^ mice to that of agonist-treated controls) for comparison in each set of experiment. After administration of HTR1a and HTR2 family agonists, the DCX increase index in hTM-DTA^iPet1^ mice was not changed relative to vehicle-treated hTM-DTA^iPet1^ mice (Fig. 6b,c). By contrast, the index was significantly reduced in hTM-DTA^iPet1^ mice treated with WAY161503, agonist of 5-HTR2c (Fig. 6b,c), suggesting that 5-HTR2c is involved in the enhanced neurogenesis in hTM-DTA^iPet1^ mice. To know whether the effect was achieved by reducing neural stem cell proliferation, we next examined BrdU-labeled cells in both control and hTM-DTA^iPet1^ mice after 3-week treatment with WAY161503. BrdU was administrated 4 times with a 2-hour interval before the mice were sacrificed. We found that the number of BrdU^+^ cells reduced dramatically in number in hTM-DTA^iPet1^ mice with no significant differences compared with controls (Fig. 6e,f). DCX^+^ cells were reduced in number as well although it was still higher in hTM-DTA^iPet1^ mice relative to control (Fig. 6g). These results suggest that impaired activation of 5-HTR2c contributes to the enhanced adult neurogenesis in hTM-DTA^iPet1^ mice.

### Altered anxiety- and depression-like behaviors, and fear memory in hTM-DTA^iPet1^ mice

**Figure 7:**
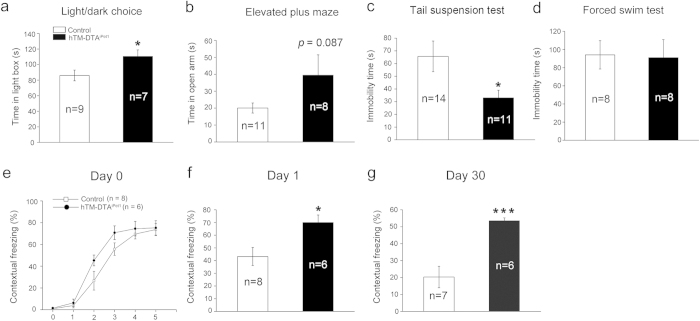
Altered anxiety- and depression-like behaviors, and fear memory in hTM-DTA^iPet1^ mice. (**a**) The time spent in the lit compartment is significantly increased in hTM-DTA^iPet1^ mice in the light/dark choice test, indicating reduced anxiety-like behaviors. Data were compared using Student’s *t*-test (*p* = 0.037). (**b**) The time spent in the open arms of the elevated plus maze is decreased in hTM-DTA^iPet1^ mice, but the difference is not statistically significant (*p* = 0.087). (**c–d**) Immobility time is significantly decreased in hTM-DTA^iPet1^ mice in the tail suspension test (**c**), but comparable between control and hTM-DTA^iPet1^ mice in the forced swim test (**d**). Data were compared using Student’s *t*-test (*p* = 0.0363 in **d**). (**e–g**) Enhanced fear memory in hTM-DTA^iPet1^ mice. Normal fear freezing is observed in hTM-DTA^iPet1^ mice on Day 0 (**e**). Data were compared using Two-way Repeated-Measures ANOVA. On Day 1 (**f**) and Day 30 (**g**), however, hTM-DTA^iPet1^ mice exhibit significantly increased contextual freezing. Data were compared using Student’s *t*-test (*p* = 0.017 in (**f)** and *p* = 8.2 × 10^−4^ in **g**). All data are presented as mean ± s.e.m. *p < 0.05; ***p < 0.001.

The enhanced adult hippocampal neurogenesis is required for fluoxetine to exert its antidepressant effect^22^. To date, there have been no published reports describing the behavioral characteristics associated with a genetic mouse model lacking the vast majority of central 5-HTergic neurons from adulthood, but having enhanced hippocampal neurogenesis. We thus set out to characterize the behavioral phenotype of hTM-DTA^iPet1^ mice, starting with anxiety-like behaviors. In the light/dark choice test, we found that hTM-DTA^iPet1^ mice spent significantly more time in the lit compartment compared to control mice (Fig. 7a). Consistently, hTM-DTA^iPet1^ mice spent more time in the open arms of the elevated plus maze than control mice did, although this difference did not reach statistical significance (Fig. 7b; *p* = 0.086). Basal depression-like behaviors were also assessed with two different tests. No difference was observed in the forced swim test, but immobility time in the tail-suspension test was significantly reduced in hTM-DTA^iPet1^ mice compared with controls (Fig. 7c,d). These results suggest that the basal anxiety- and depression-like behaviors are altered in hTM-DTA^iPet1^ mice.

Our previous study showed that *Pet1*-Cre;*Lmx1b*^flox/flox^ mice, which essentially lack all central 5-HTergic neurons from late embryonic stage, displayed enhanced contextual fear memory^23^. It has also been reported that mice with decreased adult hippocampal neurogenesis exhibit reduced fear memory^24^. We were promoted to examine whether contextual fear memory is altered in our hTM-DTA^iPet1^ mice. On the first day (Day 0), mice were given five foot shocks to acquire fear memory. Both hTM-DTA^iPet1^ and control mice showed similar increases in freezing time after each foot shock (Fig. 7e). One day (Day 1) and 30 days (Day 30) later, mice were tested to retrieve recent and remote fear memory, respectively. We found that freezing times remained high in hTM-DTA^iPet1^ mice at both time points, but decreased in control mice (Fig. 7f,g), thus demonstrating that contextual fear memory is enhanced in hTM-DTA^iPet1^ mice.

## Discussion

In the present study, we employed two approaches to genetically lower central 5-HT levels starting from adulthood. Both mouse models with lower adult central 5-HT took advantage of the inducible Cre system, in order to avoid the potential developmental defects associated with depleting central 5-HT during the development. We found that removing 5-HTergic neurons in hTM-DTA^iPet1^ mice and deleting *Tph2* in 5-HTergic neurons in Tph2^iPet1^ mice both led to significant enhancement of adult neurogenesis in the hippocampus.

Previous studies have examined hippocampal adult neurogenesis in knockout mice constitutively lacking Tph2 or 5-HTT or 5-HT receptors. Normal hippocampal neurogenesis has been reported in adult *Tph2*^−/−^ and *5-HTT*^−/−^ mice^9^,^10^. 5-HTR1a antagonists can cause a decrease in adult neurogenesis^3^, while 5-HTR1a knockout mice appear normal in this regard^22^. Central 5-HTRs are known to be functional from embryonic stages onwards, and central 5-HT plays important roles in neuronal morphogenesis and neural circuitry formation during embryonic and early postnatal development. Therefore, it is likely that these conventional knockout lines would exhibit developmental defects, and in fact growth retardation has been reported in Tph2^−/−^ mice^25^,^26^. Due to known and potential unknown secondary developmental defects, these knockouts may be suboptimal for conclusively characterizing the role of central 5-HT in adult neurogenesis.

It is well known that up-regulating 5-HT system by SSRIs enhances adult hippocampal neurogenesis^8^. In this study, we revealed that lowering 5-HT system function in adult brain by depleting 5-HTergic neurons or inactivating Tph2 expression could also enhance adult hippocampal neurogenesis. BrdU-labeled, Ki67^+^ and MCM2^+^ cells in the SGZ were all increased, and GFAP^+^/Sox2^+^ QNPs and GFAP^-^/Sox2^+^ ANPs were increased as well. In addition to increase of neural stem cells, we also found that the survival of new-born neurons generated before and after depleting 5-HTergic neurons was also enhanced in hTM-DTA^iPet1^ mice, which is consistent with the previous findings that the survival of adult-born cells is enhanced in the SGZ of mice with central 5-HT deficiency from embryonic stages including Pet1^−/−^ mice, 5-HTT^cre/+^; VMAT2^f/f^ and Tph2 KI mice^12^,^13^. Based on the data mentioned above, we think that both enhanced new-born survival and increased neural stem cell proliferation contribute to the enhancement of hippocampal neurogenesis in our mouse models.

It has been shown that 5-HTR2c antagonist increases adult hippocampal neurogenesis^27^. We revealed that chronic administraion of 5-HTR2c agonist lowered the increase of BrdU-labled cells in hTM-DTA^iPet1^ mice, suggesing that the increased adult neurogenesis in our 5-HT-deficient mice may be if not all at least partially caused by impaired activitaion of 5-HTR2c. However, althrough DCX^+^ cells were lowered in number after adminstration of 5-HTR2c agonist, it was still higher than control. These results suggest that other 5-HT receptors may be involved, although we did not detect obvouis changes in DCX^+^ cells after administration of agonist for 5-HTR1a and 5-HTR2 family. Among 5-HT receptors distributed in the dentate gyrus and hippocampal CA1-3 regions, relative expression level of 5-HTR2c is low^28^. As 5-HT is believed to be an extrinscic factor in regualting adult neurogenesis^1^,^2^,^3^, we speculate that adult 5-HT deficiency may lead to unknown alterations in activities of some neural networks which in turn result in the enhanced adult neurogenesis. Further studies are needed to examine the mechanisms underlying the enhanced hippocampal neurogensis with central 5-HT deficiency exclusively in adulthood.

Here we found that depleting over 95% of central 5-HTergic neurons in hTM-DTA^iPet1^ mice enhances adult hippocampal neurogenesis, but removing approximately 50% of them has no such effect. In our Tph2^iPet1^ mice, Tph2 is inactivated in approximately 85% of central 5-HTergic neurons, and increased adult neurogenesis is also observed, although the increasing level is less than that in hTM-DTA^iPet1^ mice. Based on these data, we can hypothesize that a threshold of central 5-HT level exists, below which hippocampal neurogenesis becomes enhanced. Different 5-HTRs show different roles in regulation of adult neurogenesis^29^. Different 5-HT receptors are expressed at varying levels within the hippocampus^28^ (http://mouse.brain-map.org), and their specific affinity to 5-HT is also variable^30^ (http://www.iuphar-db.org). It is possible that different 5-HT receptor combinations might be activated depending on whether 5-HT levels are high or low, thus affecting adult neurogenesis differentially. Enhancing 5-HT function by SSRIs is also capable of incresesing adult hippocampal neurogenesis^22^. Thus, the hippocampal neurogenesis is enhanced in the two opposite conditions by 5-HT, in which central 5-HT levels are extremely low (the present study), as well as in SSRIs-treated mice, in which 5-HT levels are high^22^ through activation different 5-HT receptors in normally-developed brain.

Our hTM-DTA^iPet1^ mice display reduced anxiety-like behaviors but enhanced contextual fear memory. Our previous results indicated that *Pet1*-Cre;*Lmx1b*^flox/flox^ mice lacking central 5-HTergic neurons at embryonic stage show similar alterations in these types of behaviors^23^ but have normal adult hippocampal neurogenesis. These results suggest that reduced anxiety-like behaviors and enhanced contextual fear memory in hTM-DTA^iPet1^ mice may be due at least in part to low levels of 5-HT itself. Running wheel training enhances the adult hippocampal neurogenesis in rodents, and physical exercises display the therapeutic effects in major depression and other behavioral deficit^31^,^32^,^33^. Lower hippocampal volume has been reported in patients suffering from depression^34^, and SSRIs-induced anti-depressant effect requires the enhanced adult hippocampal neurogenesis in mice^8^,^22^. Importantly, recent studies from *Pet1*-Cre;*Lmx1b*^flox/flox^ and Tph2 KO mice have shown that central 5-HT deficiency is not sufficient to induce depression-like behaviors in mouse^35^,^36^. Considering the important role of adult hippocampal neurogenesis in depression, it is likely that the enhanced hippocampal neurogenesis may be a key factor for lowered basal depression-like behaviors in our hTM-DTA^iPet1^ mice.

In summary, we designed and utilized two novel mouse models, in which the brain develops normally but central 5-HT is depleted in adulthood, in order to investigate the regulation of adult hippocampal neurogenesis. These models uncovered an unexpected new role for central 5-HT, demonstrating that lowering it to a certain level leads to enhanced adult hippocampal neurogenesis and this effect may be partially achieved by inactivation of 5-HTR2c.

## Methods

### Experimental animals

To deplete 5-HTergic neurons in adulthood, *Pet1*-CreER^T2^ mice^17^ were crossed with Rosa26-DTA mice^18^, and DTA^iPet1^ (i.e. *Pet1*-CreER^T2^; Rosa26-DTA) were obtained from the progeny. To block 5-HT synthesis in the adult brain, *Pet1*-CreER^T2^ mice were crossed with Tph2 floxed mice and Tph2^iPet1^ (i.e. *Pet1*-CreER^T2^; *Tph2*^flox/flox^) mice were obtained. In DTA^iPet1^ mice, tamoxifen (TM) dissolved in corn oil was administered by oral gavage for a total of three times (Day 1, 2, 3) or four times (Day 1, 2, 4, 5) beginning 2.5–3.0 months after birth. Littermates of other genotypes (wild type, Rosa26-DTA or *Pet1*-CreER^T2^) received the same TM regimen and were used as controls in each set of experiments. In Tph2^iPet1^ mice, TM was administered for a total of six times (Day 1, 2, 5, 6, 9, 10) beginning 1.5 months after birth. All experiments were performed in accordance with the Guidelines and Regulation of Laboratory Animals Used for Biomedical Studies of Shanghai, China. Animal care practices and all experiments were reviewed and approved by the Animal Committee of Tongji University School of Medicine, Shanghai, China.

### Immunohistochemistry, BrdU labeling analysis, and *in situ* hybridization

Immunohistochemical staining was performed as described in our previous studies^37^,^38^. The following primary antibodies were used: mouse anti-BrdU (1:300; Calbiochem) or rat anti-BrdU (1:1000; Accurate Chemical & Scientific Corporation), goat anti-Calretinin (CR) (1:1000; Chemicon), goat anti-doublecortin (DCX) (1:400; Santa Cruz), goat anti-NeuroD (1:400; Santa Cruz), rabbit anti-Tph2 (1:20,000) (Gutknecht *et al.*, 2008), rabbit anti-GFAP (1:500; Dako), rabbit anti-5-HTT (1:1000; gift from Dr. R.D. Blakely), rabbit anti-Ki67 (1:500; Abcam), mouse anti-MCM2 (1:1000; BD Pharmingen), goat anti-Sox2 (1:400; Santa Cruz), mouse anti-NeuN (1:1000; Chemicon) and rabbit anti-tyrosine hydroxylase (TH) (1:4000; Sigma).

For BrdU pulse labeling experiment to analyze cell proliferation, mice received 4 injections of BrdU at 50 mg/kg body weight at a 2-hour interval, and were sacrificed 2 hours after the last injection. For analysis of new-born cell survival, BrdU was injected before or after TM administration. In BrdU injection before depleting 5-HTergic neurons, BrdU was injected once daily for 3 consecutive days, followed by 4 times of TM administration; mice were sacrificed 30 days after the last TM administration. In BrdU injection after depleting 5-HTergic neurons, BrdU was injected 30 days after the last TM treatment for 3 consecutive days in the same way, and mice were allowed to survive for further 30 days after the BrdU injection. Brain sections were immersed in 0.01 M citrate buffer at 95 °C for 5 min, in 2 N HCl at 37 °C for 20 min and in 0.1 M sodium borate for 10 min, and then washed in PBS. Treated sections were immunostained with anti-BrdU antibody as described above.

AADC *in situ* hybridization was performed as described previously (Song *et al.*, 2011). Images were captured with an epifluorescence (Eclipse 80i, Nikon) or confocal (TCS SP5, Leica) microscope.

### Cell count

We counted labeled cells in every sixth section. For counting BrdU^+^, NeuroD^+^, DCX^+^, MCM2^+^, Ki67^+^, GFAP^+^/Sox2^+^, and GFAP^-^/Sox2^+^ cells in the SGZ, sections at the level of −1.34 mm to −3.52 mm from Bregma were included. For counting Tph2^+^ cells in the dorsal raphe nucleus, sections at the level of −4.72 mm from Bregma were included^17^.

### High performance liquid chromatography (HPLC)

Samples containing the cerebral cortex and hippocampus were collected and HPLC were performed as described previously^23^.

### Injection of 5-HTR agonists

Control and DTA^iPet1^ mice from 2.5-3 months old were administrated with different 5-HTR agonists. TM were injected on day 1, day 2, day 4 and day 5, while agonists were injected once daily from day 3 to day 26 on which mice were sacrificed for analysis. All agonists of 5-HTRs were purchased from Tocris and were injected by i.p. The dosage of agonists used was as follows: 8-OH DPAT (8-hydroxy-2-dipropylaminotetralin hydrobromide, agonist of 5-HTR1a), 1 mg/kg body weight; α-Methyl-5-hydroxytryptamine maleate (5-HTR2 family agonist), 0.5 mg/kg body weight; WAY161503 (5-HTR2c agonist), 10 mg/kg body weight.

### Behavioral tests

Behavioral observation was performed in adult male mice 1 month after the final TM injection. For forced swim test, mice were placed into glass cylinders (height: 24 cm; diameter: 16 cm) containing 18 cm of 23–25 °C water for 6 min. A mouse was considered to be immobile when it floated in the water, and immobility time was recorded during the last 4 min of the 6-min testing period, after 2 min of habituation.

Tail suspension test. Mice were suspended by the tail with adhesive tape (distance from tip of tail: 2 cm). Their behavior was video recorded for the duration of the 6-min testing period, with immobility time being measured during the last 4 min.

Elevated plus maze test. Mice were initially placed at the far end of one of the closed arms and allowed to freely explore the maze for 5 min. The percentage of time spent in the two open arms was then recorded.

Light/dark choice test. The light/dark choice test apparatus consisted of a small dark chamber (30 cm × 20 cm × 30 cm) connected by an opening (5 cm × 7 cm) to a larger lit chamber (30 cm × 30 cm × 30 cm). A single mouse was initially placed in one corner of the dark chamber and the percentage of time spent in the lit chamber was measured over 5 min.

Contextual fear conditioning. Contextual fear conditioning was performed as previously described^23^. FreezeFrame and FreezeView software systems were used to record and analyze freezing behaviors. On the first day (Day 0), mice were given five foot shocks (0.7 mA, 2s) at 2 min intervals during which the mice were able to move freely. The percentage of freezing time was measured during each inter-shock interval. On the second day (Day 1) and one month later (Day 30), mice were placed back in the box for 11 min without receiving any foot shocks, and freezing time was measured to test contextual fear memory.

### Statistical analysis

All samples passed both the Shapiro-Wilk normality test and equal variancetest except for 5-HT level in Fig. 1d, BrdU^+^ cell numbers in Fig. 3d and BrdU^+^ cells in BrdU after TM groups in Fig. 4d–e. Comparisons were performed using the Student’s *t*-test, one-way ANOVA with *post hoc* Tukey test or Mann-Whitney rank sum test (for samples which didn’t pass normality or equal variance test). All data are presented as mean ± s.e.m. *P* values of less than 0.05 were considered statistically significant.

## Additional Information

**How to cite this article**: Song, N.-N. *et al.* Reducing central serotonin in adulthood promotes hippocampal neurogenesis. *Sci. Rep.* **6**, 20338; doi: 10.1038/srep20338 (2016).
